# Single-cell RNA sequencing reveals special basal cells and fibroblasts in idiopathic pulmonary fibrosis

**DOI:** 10.1038/s41598-024-66947-5

**Published:** 2024-07-09

**Authors:** Chengji Jin, Yahong Chen, Yujie Wang, Jia Li, Jin Liang, Shaomao Zheng, Lipeng Zhang, Qiaoyu Li, Yongchao Wang, Fayu Ling, Yongjie Li, Yu Zheng, Qiuli Nie, Qiong Feng, Jing Wang, Huiling Yang

**Affiliations:** 1 Department of Respiratory Medicine, The Second Affiliated Hospital, Hainan Medical University, Haikou, 570100 China; 2 NHC Key Laboratory of Tropical Disease Control, Hainan Medical University, Haikou, 571199 China; 3 School of Pharmacy, Guangdong Medical University, Dongguan, 523808 China; 4 The Second Affiliated Clinical College, Hainan Medical University, Haikou, 570100 China; 5 Department of Rheumatology and Immunology, The Second Affiliated Hospital, Hainan Medical University, Haikou, 570100 China; 6 Singleron Biotechnologies, Yaogu Avenue 11, Nanjing, 211800 China; 7 Department of Thoracic Surgery, The Second Affiliated Hospital, Hainan Medical University, Haikou, 570100 China

**Keywords:** Idiopathic pulmonary fibrosis, Single-cell RNA-seq, Basal cells, Fibroblasts, Extracellular matrix, Cell–cell communications, Cell biology, Medical research, Pathogenesis

## Abstract

Idiopathic pulmonary fibrosis (IPF) is the most predominant type of idiopathic interstitial pneumonia and has an increasing incidence, poor prognosis, and unclear pathogenesis. In order to investigate the molecular mechanisms underlying IPF further, we performed single-cell RNA sequencing analysis on three healthy controls and five IPF lung tissue samples. The results revealed a significant shift in epithelial cells (ECs) phenotypes in IPF, which may be attributed to the differentiation of alveolar type 2 cells to basal cells. In addition, several previously unrecognized basal cell subtypes were preliminarily identified, including extracellular matrix basal cells, which were increased in the IPF group. We identified a special population of fibroblasts that highly expressed extracellular matrix-related genes, *POSTN, CTHRC1, COL3A1, COL5A2*, and *COL12A1*. We propose that the close interaction between ECs and fibroblasts through ligand–receptor pairs may have a critical function in IPF development. Collectively, these outcomes provide innovative perspectives on the complexity and diversity of basal cells and fibroblasts in IPF and contribute to the understanding of possible mechanisms in pathological lung fibrosis.

## Introduction

Idiopathic pulmonary fibrosis (IPF) is the predominant form of idiopathic interstitial pneumonia and is distinguished by excessive deposition of extracellular matrix (ECM)^1,2^. With the aging global population, the incidence of IPF has been steadily increasing in recent years, and the prognosis of patients is poor, usually dying within 2.7–3.0 years of the initial diagnosis^3,4^. Although antifibrotic drugs, such as nintedanib and pirfenidone, have been approved for IPF treatment, their therapeutic effects remain unsatisfactory^5,6^. Patients eventually require lung transplantation; however, there is a lack of donors and severe immune rejection responses^7,8^. Therefore, a better understanding of the diversity of cells in IPF and their underlying pathogenesis is urgently required to explore new treatments.

Despite extensive research over the past decades using in vitro techniques and several animal models^9–11^, the pathobiological mechanisms of IPF remain unclear. Genomic data based on IPF lung tissue have provided insights into the diagnosis and prognosis of IPF^12,13^; however, the complexity of the tissue and heterogeneity of cell types have made it difficult to identify the key factors underlying pathogenesis. Recently, single-cell RNA sequencing (scRNA-seq), which may surpass the constraints of bulk tissue-based analyses, has allowed the study of gene expression in individual cells^14,15^. This technique has been used to show that pathological pulmonary fibrosis is caused by changes in cellular diversity^16^. Other studies have validated the feasibility of this technology using alveolar macrophages^17^ and airway epithelial^18^, ciliated^19^, and club cells^20^.

To obtain a more comprehensive and in-depth detailed comprehension of the cell types, states, and relationships of differentiation in the lungs of patients with IPF, this study used scRNA-seq to sequence single-cell suspensions originating from five IPF and three control lungs. We identified diverse distinct basal cells and fibroblast cellular subpopulations. Additionally, we found ECM basal cells (ECM_BCs) and ECM fibroblasts (ECM_FIB) that had not been previously described, which could generate components of the ECM, such as collagen.

## Results

### Summary of the single-cell expression atlas in normal lung and IPF tissues

**Figure 1:**
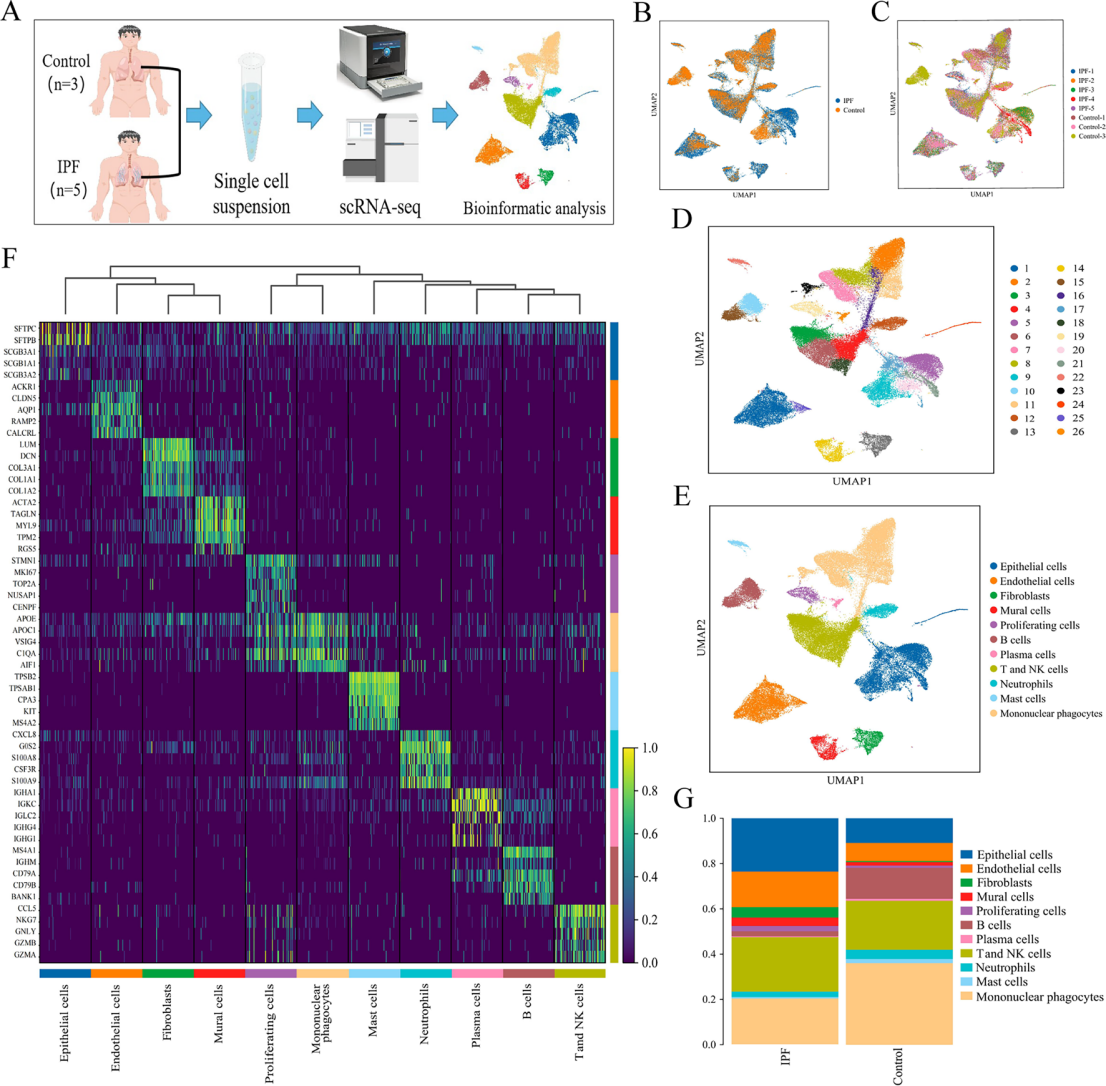
Single-cell landscape of IPF and control lungs. (**A**) Overview of experimental design and workflow. (**B**,**C**) UMAP plot displaying the cell distribution in tissue and patient source. (**D**) UMAP plot of all cells profiled, including 26 cell clusters. (**E**) UMAP plot displaying the cell types in the two groups; cell types are represented by various colors. (**F**) Heatmap displaying the top five marker genes for each cell type. (**G**) Bar plot of cell proportions in the IPF and control groups. IPF, idiopathic pulmonary fibrosis.

In total, eight lung tissue specimens were gathered from five patients diagnosed with IPF who underwent lung transplantation and three patients undergoing surgery for a lung nodule that was ultimately found to be benign. The participants’ clinical information is shown in Table S1. The lung tissue's single-cell suspensions were sequenced using a 10× Genomics platform (Fig. 1A). Following quality control and filtration, 75,613 cells were used for subsequent analysis, of which 43,857 cells were from patients with IPF and 31,756 were from healthy controls (Fig. 1B and C). Following the removal of batch effects across many samples, the process of cell classification, and the identification of marker genes, a total of 26 distinct cell clusters were discovered. These clusters were then displayed using the UMAP dimensionality reduction approach (Fig. 1D). We classified the 26 clusters into 11 cell types (Fig. 1E) based on previous studies of classical cell markers^16,17,21^. These included ECs, proliferating cells, three stromal cell types (endothelial cells, fibroblasts, and mural cells), and six types of immune cells (mononuclear phagocytes, T and NK cells, B cells, neutrophils, mast cells, and plasma cells). Figure 1F displays a heatmap with the top five marker genes for each cell type. Gene expression was unique in each cell type, demonstrating the validity of our clustering and annotations.

The percentages of cell types in the two groups were plotted to avoid errors in the comparison owing to discrepancies in the number of cells (Fig. 1G). The proportions of epithelial, endothelial, fibroblast, and mural cells were considerably elevated in the IPF group opposed to the control group (Fig. 1G and Table S2). We, therefore, postulated that these cell types have a key function in the IPF pathogenesis and performed further analyses of ECs and fibroblasts.

### ECs phenotypes change significantly between the IPF and control groups

**Figure 2:**
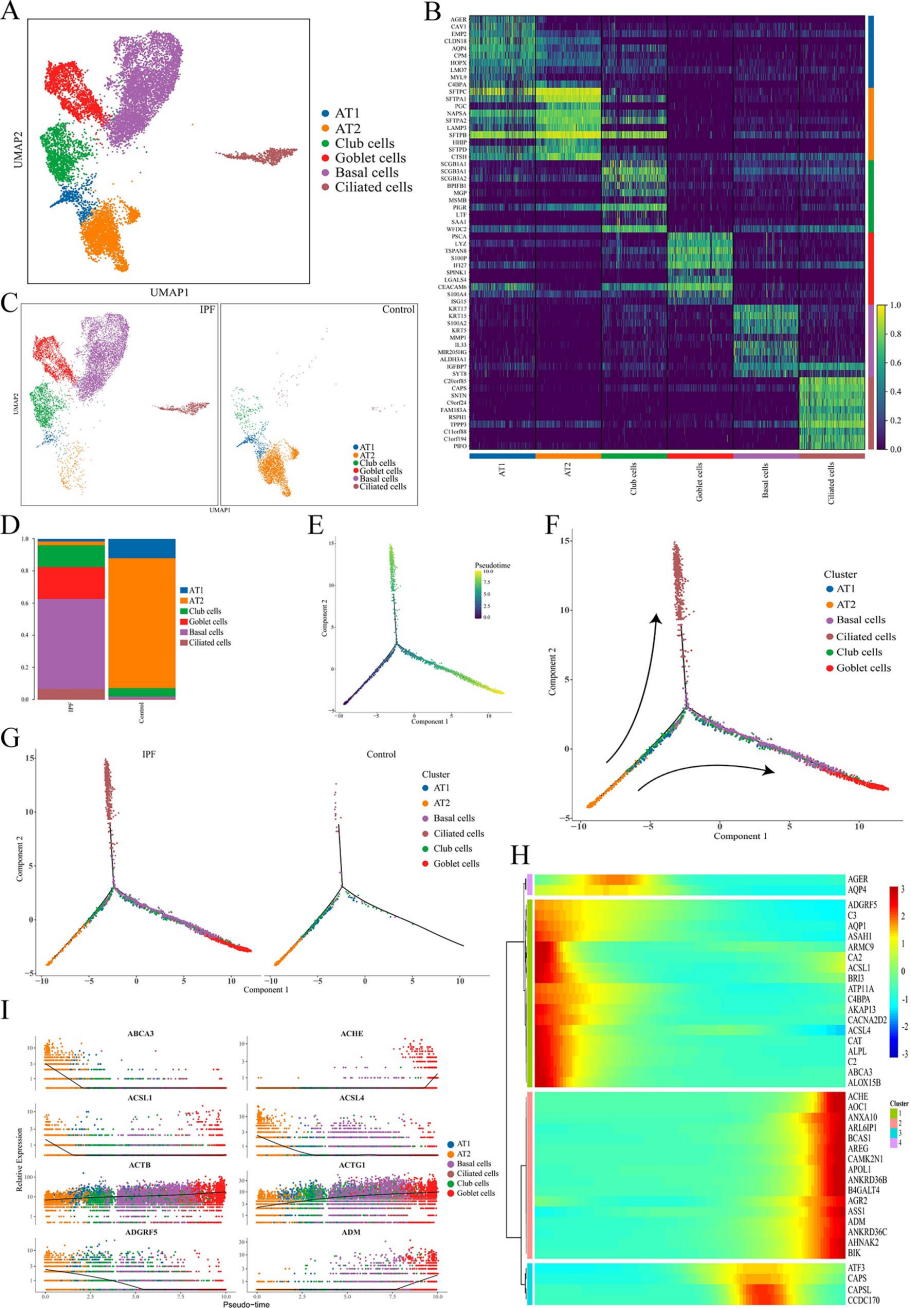
Difference in ECs subtype in IPF and control groups. (**A**) UMAP plot visualizes six subclusters of ECs. (**B**) Heatmap of the top 10 marker genes per cluster. (**C**) UMAP plots of six subclusters in IPF and control groups. (**D**) Bar plots of the proportion of ECs subtypes in the two groups. (**E**,**F**) Pseudotime trajectory analysis plotting the differentiation pattern of AT1, AT2, basal, goblet, ciliated, and club cells. (**G**) Trajectory plots display ECs subtype differentiation in IPF and control groups; cells are colored by cell types. (**H**) Heatmap of representative pseudotime-dependent genes. (**I**) Gene expression dynamics: changes in the top eight genes are displayed. The x-axis represents pseudotime, and the y-axis represents the expression level of each gene. IPF, idiopathic pulmonary fibrosis.

We identified relatively common lung EC populations, encompassing alveolar type 1 (AT1) and type 2 (AT2), basal, goblet, ciliated, and club cells (Fig. 2A), with each population expressing its respective marker gene (Fig. 2B). The UMAP results showed that the number of basal, goblet, ciliated, and club cells in the IPF lung tissue elevated substantially, whereas the number of AT1 and AT2 cells decreased compared to those in the controls (Fig. 2C). The outcomes were in line with the findings from the examination of cell proportions (Fig. 2D). The results of pseudotime analysis indicated that AT2 cells were located at the initiation site of the differentiation trajectory. AT1, basal, and club cells were positioned at later developmental stages than AT2 cells and goblet and ciliated cells were at the end of the differentiation trajectory (Figs. 2E,F, and S1). ECs with a missing distribution in the control group were identified as basal, goblet, and ciliated at the end of the differentiation trajectory (Fig. 2G). The outcomes were in agreement with the proportional distribution of EC subclusters results (Fig. 2D). The genes in the ECs were clustered into four modules depending on significant differences in their expression along the pseudotime trajectory (Fig. 2H). Module 1 (green) contained genes whose expression level reduced along the pseudotime axis; module 2 (red) contained genes whose expression was concentrated at the end of the pseudotime axis; module 3 (blue) contained genes whose expression started to increase in the posterior segments of pseudotime axis; and in module 4 (purple), the genes showed an initial rise in expression level, followed by a subsequent fall along the pseudotime axis. Expression patterns of the top eight genes, ABCA3, ACSL1, ACSL4, ADGRF5, ACHE, ADM, ACTB, and ACTG1 (Fig. 2I), were in accordance with the findings of the heatmap displayed in Fig. 2H.

### Classification and identification of basal cells in IPF lung tissue

**Figure 3:**
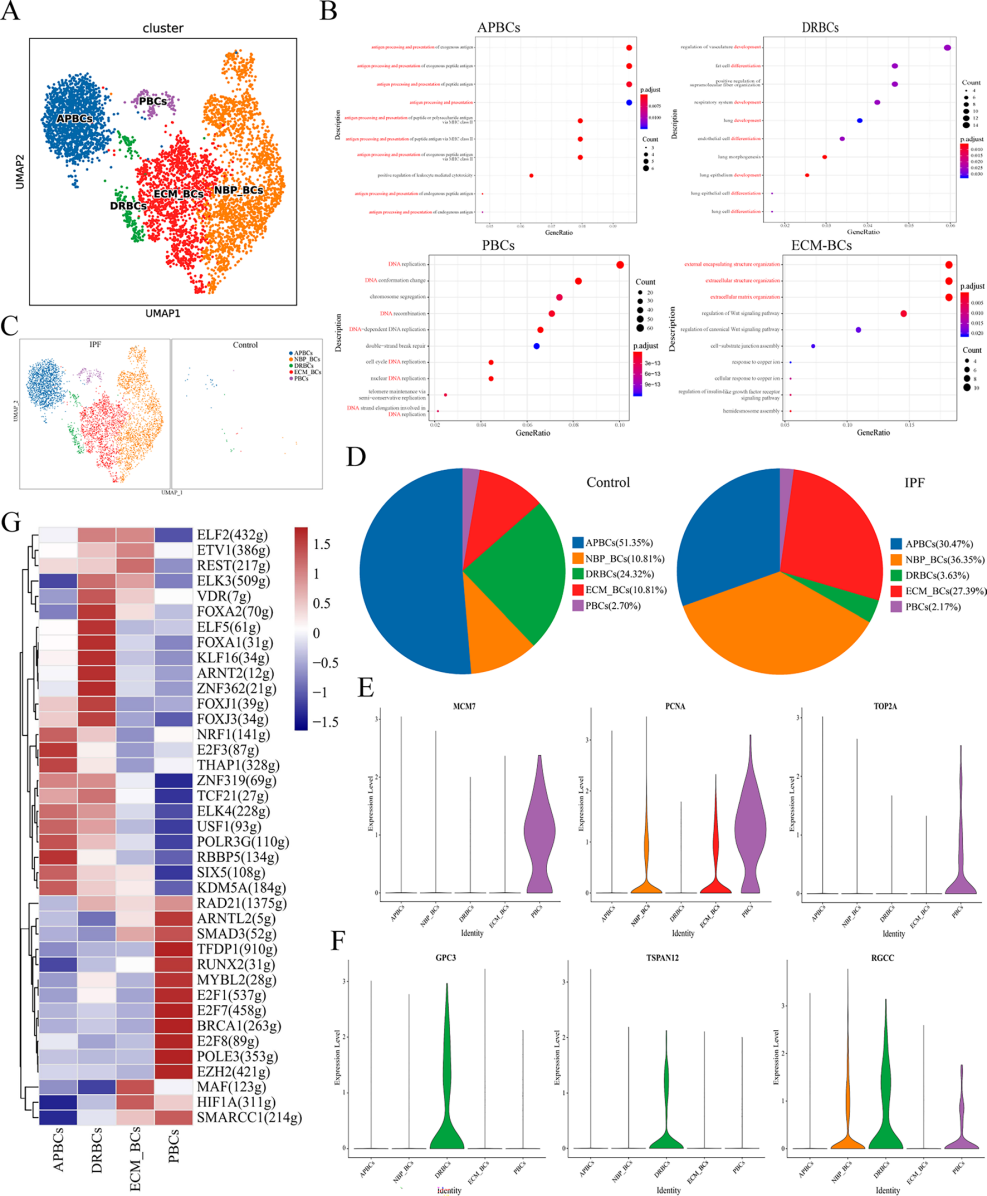
Classification and identification of basal cells. (**A**) UMAP plot displaying subsets of basal cells. (**B**) Results of biological process (BP) enrichment analysis. (**C**) UMAP plots of five subclusters of basal cells in IPF and control groups. (**D**) Pie chart showing the proportion of different subpopulations. (**E**,**F**) Violin plots of differentially expressed genes in the different subpopulations. (**G**) Heatmap showing differences in transcription factor (TF) activity across subclusters of basal cells scored by SCENIC.

We focused on the basal cells for a detailed analysis, based on the large difference in their numbers between the IPF and control groups (Fig. 2D). After dimension reduction, basal cells were further classified into five subtypes (Fig. 3A). Four basal cell types exhibited unique biological processes (BPs) (Fig. 3B); therefore, they were denoted antigen-processing and presentation basal cells (APBCs), development-and differentiation-related basal cells (DRBCs), proliferating basal cells (PBCs), and ECM basal cells (ECM_BCs). The last type had no distinctive function and were denoted none biological processes basal cells (NBP_BCs). The expression patterns for the marker genes of each subtype are shown in Fig. S2 and Table S3. Figure 3C and Table S4 show that there were very few BCs overall in the control group compared with those in the IPF group. The proportions of ECM_BCs and NBP_BCs increased, whereas the proportions of APBCs and DRBCs decreased in the IPF lung tissues (Fig. 3D). This illustrates that the two groups showed high heterogeneity in both cell types and the numbers of BCs. To validate the biological functions of these subsets, we analyzed proliferation-associated genes. The results suggested that MCM2–MCM7, PCNA, TOP2A, and CENPX were highly expressed in PBCs compared to the other cell types (Figs. 3E and S3). In addition, genes related to development and differentiation, TSPAN12, GPC3, and RGCC, were only highly expressed in DRBCs (Fig. 3F). SCENIC analysis was applied to predict TFs and their corresponding target genes among the cell types. This identified a set of TFs associated with the biological signatures of distinct basal cell subtypes (Fig. 3G). For example, E2F1, E2F7, and E2F8 were activated in PBCs to promote cell proliferation and regulate the cell cycle^22–24^. Additionally, SMAD3, which is the downstream gene of TGF-β, was activated in the ECM_BCs of the IPF group.

### Classification and identification of fibroblasts in IPF lung tissue

**Figure 4:**
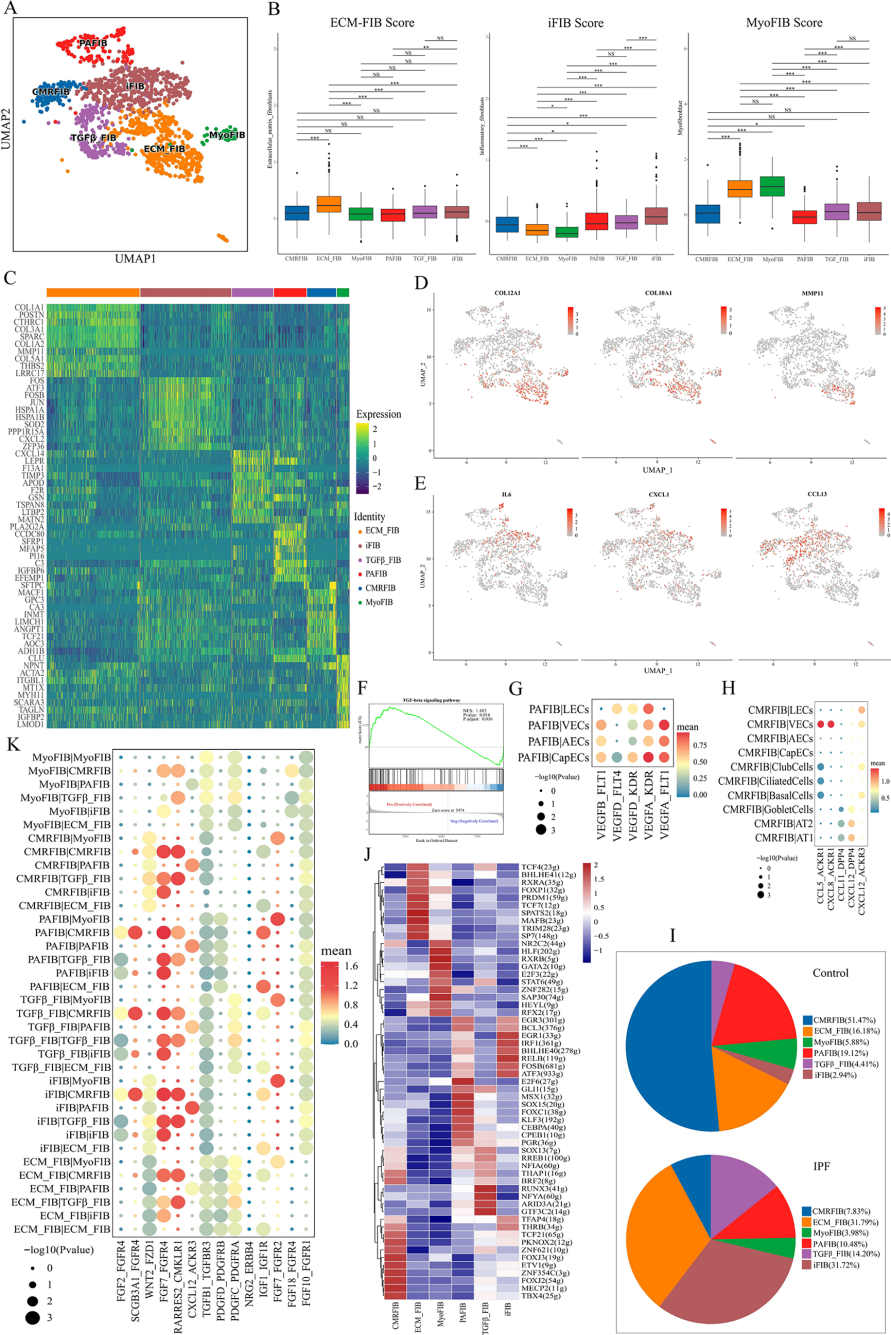
Classification and identification of fibroblasts. (**A**) UMAP plot displaying subclusters of fibroblasts. (**B**) UCell scores for ECM_FIB, iFIB, and MyoFIB. (**C**) Heatmap of the top 10 DEGs in each cluster. (**D**,**E**) Feature plots for the expression of signature genes in ECM_FIB and iFIB. (**F**) Gene set enrichment analysis for TGF-β signaling pathway. (**G**) Bubble plot of receptor–ligand pairs for communication between PAFIB and the subsets of endothelial cells. (**H**) Bubble plot of receptor–ligand pairs for communication between CMRFIB and the subsets of endothelial cells and ECs. (**I**) Pie chart of the proportion of fibroblast subtypes in the two groups. (**J**) Heatmap showing differential TF activity across different subclusters in fibroblasts identified by SCENIC. (**K**) Cell–cell communication analysis in the subclusters of fibroblasts. CMRFIB, cell migration-related fibroblast; ECM_FIB, extracellular matrix fibroblast; iFIB, inflammatory fibroblast; MyoFIB, myofibroblast; PAFIB, pro-angiogenesis fibroblast.

Focusing on the fibroblasts, we identified six cell populations (Fig. 4A). Each subpopulation showed a unique biological process (Fig. S4) and was designated as ECM fibroblasts (ECM_FIB), inflammatory fibroblasts (iFIB), TGF-β signaling pathway-related fibroblasts (TGFβ_FIB), pro-angiogenesis fibroblasts (PAFIB), cell migration-related fibroblasts (CMRFIB), and myofibroblasts (MyoFIB). The biological functions of ECM_FIB, iFIB, and MyoFIB were validated using UCell scores (Fig. 4B). The heatmap showing the top 10 DEGs for each fibroblast subtype is shown in Fig. 4C. ECM_FIB was exhibited with elevated expression of the cell markers POSTN, CTHRC1, and LRRC17; iFIB highly expressed FOS, ATF3, and FOSB; TGFβ_FIB showed high CXCL14 and F13A1 expression; PAFIB specifically expressed the cell markers SFRP1, MFAP5, and PLA2G2A; CMRFIB highly expressed SFTPC, GPC3, and INMT; and MyoFIB specifically expressed the cell markers ACTA2, MYH11, and NPNT. Figure S5 and Table S5 display the cellular populations' cluster-specific marker gene expression patterns. We then validated the biological functions of these subsets from different perspectives. ECM _FIB highly expressed COL12A1, COL10A1, and MMP11, in addition to overexpressing COL1A1, COL3A1, COL1A2, and COL5A1 (Fig. 4C and D). The specific expression of IL-6, CXCL1, and CCL13 in iFIB was also validated in the feature plot (Fig. 4E). The results of GSEA showed that the TGF-β signaling pathway was activated in TGFβ_FIB (Fig. 4F). In addition, we found that the ligand–receptor pairs VEGF_FLT and VEGF_KDR between PAFIB and endothelial cells remained in the active state (Fig. 4G). CMRFIB interacted with endothelial and ECs through ligand–receptor pairs related to chemokines (Fig. 4H). The findings together confirm the validity of our classification and identification of the fibroblasts. We analyzed the variations in the proportions of these cells between control and IPF groups. The proportions of ECM_FIB, iFIB, and TGFβ_FIB increased, whereas the proportions of PAFIB and CMRFIB decreased in the IPF group as opposed to those in the control (Fig. 4I). SCENIC analysis revealed a potential regulatory network of TFs across the fibroblast subtypes (Fig. 4J). TCF4, MAFB, PRDM1, TCF7, SPATS2, and SP7 were found as potential TFs responsible for the gene expression differences observed in ECM_FIB. CellPhoneDB analysis was performed to detect interactions between the subclusters of fibroblasts and the ligand–receptor pair interactions (Fig. 4K). In addition to the recognized ligand–receptor pairs, PDGF–PDGFR and FGF–FGFR, we also discovered new pairs, such as SCGB3A1–FGFR4, RARRES2–CMKLR1, and CXCL12–ACKR3.

### Bipolar differentiation of AT2 cells and the interaction between ECs and fibroblasts

**Figure 5:**
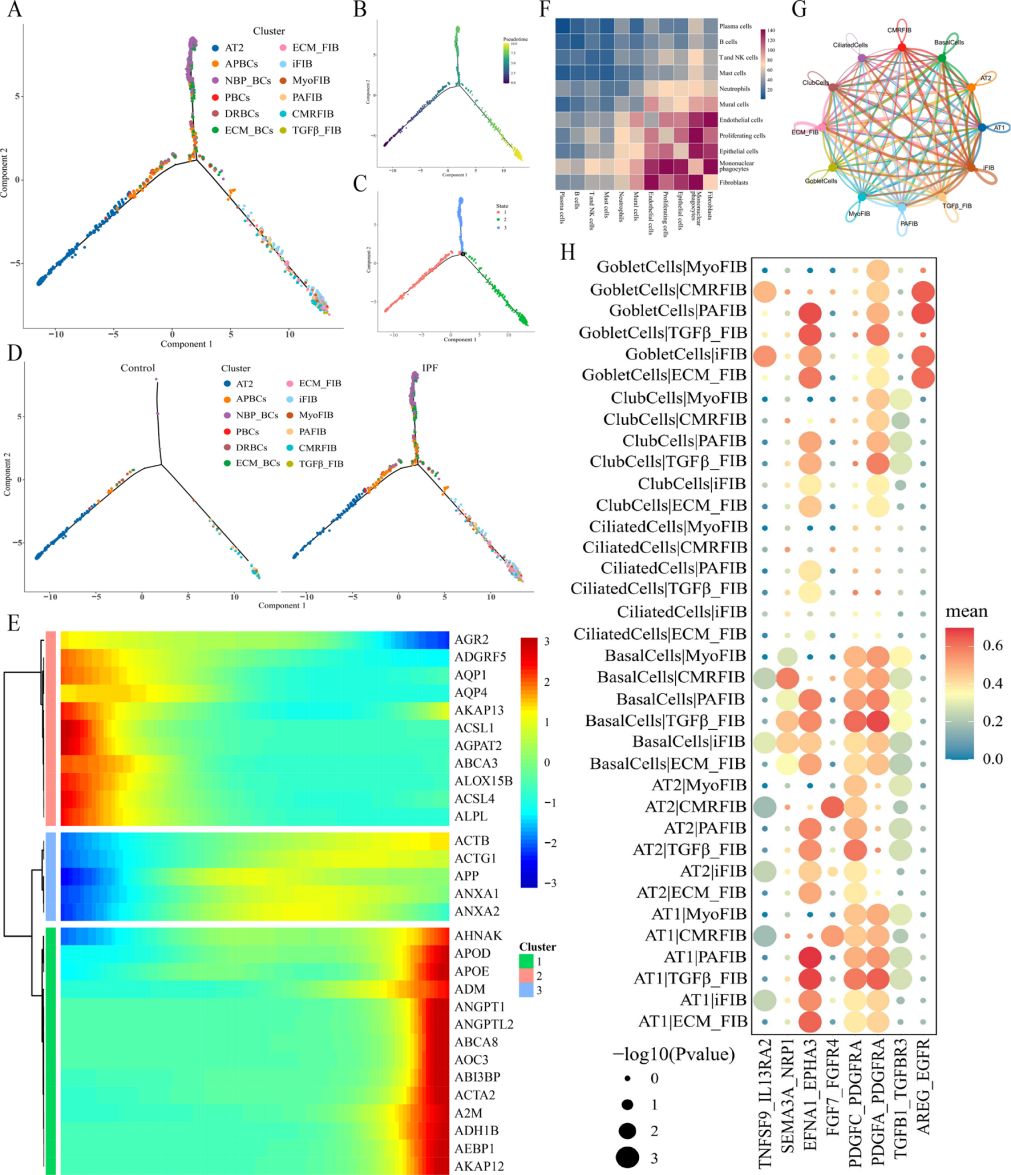
The interaction between ECs and fibroblasts. (**A**,**B**) Pseudotime trajectory analysis of AT2 cells, BCs, and FIBs. Cells are colored by pseudotime. (**C**) Trajectory plots show that AT2 cells, BCs, and FIBs are separated into three cell states. (**D**) Trajectory plots showing AT2 cell differentiation in control and IPF groups. Cells are colored by subtypes. (**E**) Heatmap showing pseudotime-dependent differentially expressed gene clusters for BCs, FIBs, and AT2 cells. (**F**) Heatmap of the communication strength between major cells. (**G**) Network diagram of the weight and number of receptor–ligand interactions between ECs subtypes and fibroblast subsets. (**H**) Bubble plot of the receptor–ligand pairs for ECs and fibroblasts. ECM_BC, extracellular matrix basal cell; ECM_FIB, extracellular matrix fibroblast.

To investigate the origin of BCs and FIBs further in the control and IPF groups, we performed a single-cell pseudotime trajectory analysis. The results showed that BCs and FIBs potentially originated from the same AT2 cells but via different differentiation trajectories (Fig. 5A and B). Meanwhile, BCs were also in the transitional stage of differentiation from AT2 to FIBs(Figs. 5A and S6). The IPF group comprised more state 2 cells (FIBs) and state 3 cells (BCs) than those in the control group (Fig. 5C and D). The pseudotime-dependent genes were classified into three gene clusters (cluster1–cluster3) based on their patterns of expression (Fig. 5E). For example, we identified that the gene sets of cluster 1 (green) have a key function in the FIBs fate.

In order to conduct a more thorough examination of the interaction between fibroblasts and other cells in the lung tissue throughout the IPF development, we conducted a cell-to-cell communication analysis and found that the communication between fibroblasts and mononuclear phagocytes, endothelial, proliferating, and ECs was similar (Fig. 5F). We further analyzed the cell–cell communication between ECs and fibroblasts (Fig. 5G). The results showed that signal crosstalk existed between ECs subtypes and other fibroblasts (Fig. 5H), and their communication primarily focused on the augmented binding of three ligand receptors, EFNA1–EPHA3, PDGFC–PDGFRA, and PDGFA–PDGFRA. These results suggest that a complex interaction exists between ECs and fibroblasts.

### Validation of newly identified ECM_BCs and ECM_FIBs

**Figure 6:**
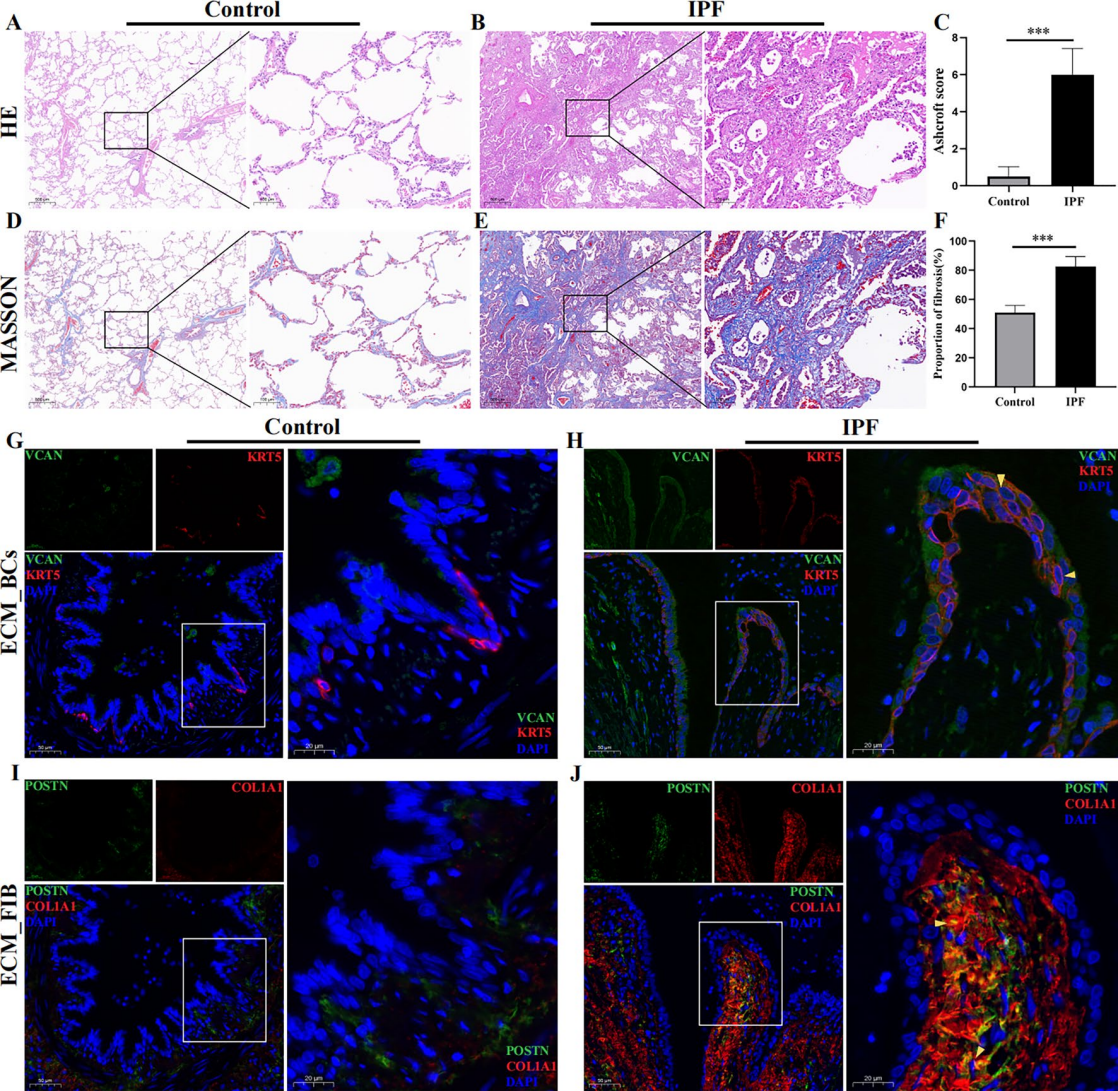
Immunofluorescence staining for ECM_BCs and ECM_FIB. (**A**,**B**) The representative histological images of human lung tissue stained by HE; Scale bar: 100 μm. (**C**) Ashcroft score of lung tissue sections (n = 8). (**D**,**E**) Typical images of lung tissue of human were obtained by Masson staining; Scale bar: 100 μm. (**F**) Percentage of Masson staining sections with fibrosis (n = 8). (**G**–**J**) Multiplexed immunofluorescence staining for ECM_BCs(KRT5+/VCAN+) and ECM_FIB(COL1A1+/POSTN+); (n = 5).

Firstly, HE and Masson staining of lung tissue sections were performed. HE staining showed that the alveolar septum was destroyed and broadened in IPF group, accompanied by the extensive infiltration of inflammatory cells, whereas the alveolar structure of control group was clear and complete (Fig. 6A and B). The Ashcroft score reaveled that the degree of lung fibrosis was higher in the IPF group (Fig. 6C). Meanwhile, the results of Masson staining revealed that massive collagen deposition were observed in the IPF lung tissue compared with control group (Fig. 6D–F). Subsequently, in order to determine the presence of ECM_BCs and ECM_FIB, we performed immunofluorescence staining using specific markers (Tables S3 and S5) enriched in these two subtypes. ECM_BCs(KRT5^+^/VCAN^+^, Fig. 6H, yellow arrows) and ECM_FIB(COL1A1^+^/POSTN^+^, Fig. 6J, yellow arrows) were found in subepithelial regions around airways in IPF and rarely around airways in control lungs(Fig. 6G–J). Furthermore, COL1A1 was highly expressed below ECM_BCs (Fig. 6H and J).

## Discussion

Despite significant advancements made in recent decades, our understanding of the fundamental processes that cause IPF is still limited. In this paper, we established a single-cell transcriptome atlas of IPF lung and found an elevation in the number of stromal cells, including fibroblasts and endothelial, mural, and ECs in the IPF group. Dysregulated crosstalk between the epithelial and stromal cells, which are an important source of ECM proteins^25^, is ubiquitous in IPF^26^. Prior research has shown that ECs and fibroblasts influence the risk of IPF^27,28^. Our subsequent analyses focused on ECs and fibroblasts and identified several key cell subtypes. We observed an elevated quantity of basal cells and a reduced quantity of AT2 cells among the ECs. In addition to highly expressing the marker genes KRT5 and KRT15^29^, basal cells also specifically overexpressed KRT17 and S100A2 (Fig. 2B). A prior investigation validated the existence of KRT5+/KRT17+ basal-like cells mostly situated in regions of severe tissue remodeling in the lungs of individuals with IPF^30^. Habermann et al.^31^ identified a KRT5−/KRT17+ cell population that can produce ECM in IPF peripheral lung tissue. Furthermore, Huang et al.^32^ found that pulmonary fibrosis can be alleviated through inhibition of epithelial-mesenchymal transition by downregulating S100A2. We speculate that the quantitative change in AT2 and basal cells is linked to the differentiation from AT2 to basal cells, as shown in the results of the pseudotime analysis. This result is similar to that of some recent reports suggesting differentiation trajectories from AT2 not only to AT1^33^ but also to KRT5^+^ basal cells through alveolar-basal intermediates (ABIs)^34^ and AT2 cells may be derived from respiratory airway secretory cells^35^. Notably, ACHE and ADM, which played key roles at the end of the pseudotime axis, are closely associated with wound healing^36,37^. Fibrosis is defined as the excessive deposition of ECM during wound healing; therefore, we believe that basal cells contribute to the pathological process of lung fibrosis.

Basal cell subsets were sorted based on the important role of basal cells in IPF. We identified four subpopulations of basal cells with unique biological processes, namely APBCs, DRBCs, PBCs, and ECM_BCs. Like dendritic cells and macrophages, APBCs had an antigen-presenting function, which extends our knowledge of the role of basal cells. DRBCs are centrally involved in lung development and showed high expression levels of GPC3, TSPAN12 and RGCC, which have been revealed to be closely linked to the development and differentiation^38–40^. We also identified PBCs that highly expressed genes related to DNA replication and cell proliferation, such as PCNA and TOP2A^41,42^. Furthermore, all members of the minichromosome maintenance (MCM2–MCM7) family, which is responsible for DNA replication^43^, were overexpressed in PBCs. Similar PBCs were manifested in the paper performed by Carraro et al.^44^, however, the identification of other subtypes of basal cell is less consistent with our results. ECM_BCs were mainly contributed to ECM organization and regulation of the Wnt signaling pathway. There was a substantial variation in the number and proportion of subsets between the IPF and control groups. A decrease in the percentages of APBCs in the IPF group, resulting in a reduced antigen-presenting capability, may also contribute to repeated cycles of injury and repair when inflammation occurs. Furthermore, the increased number of ECM_BCs in the IPF group would potentially lead to excessive deposition of ECM, providing robust evidence for the putative role of lung ECs producing pathologic ECM. The percentage of DRBCs in the IPF group was lower than that of the controls, and an enrichment in the transcription factors FOXA2 and FOXJ1, which have been reported to be strongly associated with lung development and cell differentiation, was observed^45,46^. Therefore, we presume that DRBCs also play an important role in IPF. Collectively, these outcomes provide a thorough comprehension of the potential role of basal cells in IPF.

Fibroblast populations are integral to the discussion of IPF. Despite notable progress in the description of pathogenic mechanisms of IPF^47^, there is less knowledge on the variety of fibroblast subsets in the lungs of individuals with IPF. In this study, we identified six fibroblast subtypes. In addition to MyoFIB, which is well recognized and has a central function in the pathogenesis of IPF^48^, we also identified ECM_FIB. ECM_FIB which marked by POSTN is an entirely novel subtype that has not previously been reported and is characterized by overexpression of typical ECM genes, including COL1A1, COL3A1, and COL1A2, but lacks the generally recognized MyoFIB marker ACTA2. This is similar to earlier descriptions by Tsukui et al.^49^ on Cthrc1+ fibroblasts, but CTHRC1 can't discriminate well between ECM_FIB and MyoFIB as shown in Fig. S5 of our results. Moreover, TCF4 and MAFB, which are highly enriched in ECM_FIB, are associated with renal fibrosis and epithelial–mesenchymal transition, respectively^50,51^. The number of ECM_FIB substantially elevated in the IPF group as opposed to that in control, whereas the MyoFIB levels did not change substantially between the two groups. Furthermore, the IPF group had a considerably greater proportion of iFIB than that in the control group, illustrating the critical importance of iFIB. This theory is supported by two studies. Bolourani et al.^52^ demonstrated that eCIRP contributes to pulmonary fibrosis by inducing inflammatory fibroblasts in a TLR4-dependent manner, and Xiong et al.^53^ demonstrated that the gene set, which is enriched in patients with Crohn's disease and high levels of fibrosis is linked to inflammatory fibroblasts. The main role of CMRFIB is recruiting endothelial and ECs via chemokines, and the percentage of this subtype in the IPF group was reduced as opposed to control. Therefore, we postulate that CMRFIB is involved in normal tissue repair, but not excessive repair, by recruiting endothelial and ECs. It is well known that TGF-β is closely associated with pulmonary fibrosis, and our outcomes manifested that the proportion of TGFβ_FIB in the IPF group was higher as opposed to the control group. Different with our findings, Wang et al.^54^ identified four subclusters of fibroblasts in Fibrotic hypersensitivity pneumonitis (FHP), including ACTA2^high^, COL1A1^high^, TCF21^high^, and PLA2G2A^high^ fibroblasts. Among these, ACTA2^high^ and PLA2G2A^high^ fibroblasts are similar to MyoFIB and PAFIB in our study. However, we do not share the identification of COL1A1^high^ fibroblast, because COL1A1 is typical marker genes in fibroblasts. It should not be used to identify subclusters of fibroblasts. This study also demonstrated that there is a close interaction among each subtype of fibroblasts through ligand–receptor pairs. PDGFs–PDGFRs, FGFs–FGFRs, SCGB3A1–FGFR4, RARRES2–CMKLR1, and CXCL12–ACKR3 were found to be enriched, with high expression in the cell–cell communication analysis. Among them, PDGFR and FGFR, which have been used to develop nintedanib, are closely related to pathogenesis of IPF^55,56^. Therefore, we suggest that the other three ligand–receptor pairs have the potential to be novel therapeutic targets for IPF. In summary, we identified a new fibroblast subtype expressing pathologic ECM and demonstrated that distinct fibroblast groups may also play an important role in IPF. This research offers novel perspectives on the involvement of different fibroblast cell types in the development of IPF.

Currently, the role of basal cells and fibroblasts in IPF has been demonstrated in multiple studies^57,58^, but the origin of these two classes of cells has been an area of controversy. Our study investigated the origin of basal cells and fibroblasts. Pseudotime trajectory analysis implied that these two cell types both originate from AT2 cells. Three gene clusters were identified according to their modes of expression, and the gene sets of cluster 1 were shown to be involved in the regulation of the differentiation of AT2 to fibroblasts. Among them, AEBP1 has been shown to activate fibroblasts in hypertrophic and failing human hearts^59^. Additional research is necessary to ascertain the roles of the other genes in cluster 1. The results of communication analysis between ECs and fibroblasts indicated that all the subtypes of ECs closely interact with fibroblasts through ligand–receptors, with the exception of ciliated cells. The relationship between the highly enriched ligand–receptor pair of EFNA1–EPHA3 and IPF has not previously been reported and provides a new direction for further investigation of the pathogenesis of IPF. Moreover, the key subsets of basal cells and fibroblasts, such as ECM_BCs and ECM_FIB, have been shown to be present around the airway with large amount of collagen deposition by immunofluorescence. It demonstrates the important role of this two subsets in IPF and more follow-up experiments need to be performed to explore deep molecular mechanism in the future.

The current investigation has many constraints. Our study had a relatively low number of samples, and these were collected at the time of lung transplantation, therefore representing an advanced disease state of IPF. The presence of these significant alterations in the first stages of the illness remains uncertain. Future research with large sample sizes and early disease stages are required to investigate these findings further and their potential implications for the diagnosis and treatment of IPF.

In summary, our study provides evidence of the fibrosis ecosystem heterogeneity between patients with IPF and healthy controls in terms of cell types, subtypes, EC developmental trajectories, and the crosstalk between ECs and fibroblasts. We found that ECM_BCs and ECM_FIB may be associated with the development and progression of IPF. Our results facilitate a deeper understanding of the mechanisms associated with the occurrence of IPF and may assist in the development of more effective therapeutic targets and biomarkers in IPF patients.

## Materials and methods

### Collection of human samples

Healthy control lungs (n = 8) were acquired from patients having surgery for a lung nodule that was ultimately found to be benign. Tissues from individuals with IPF who were undergoing lung transplantation were obtained (n = 8). The IPF diagnosis was established depending on the criteria specified by the American Thoracic Society/European Respiratory Society^60^. Written informed permission was obtained from all donors, and this research received approval from the Ethics Committee of the Second Affiliated Hospital of Hainan Medical College (LW2023162). The patient data is included in Supplementary Table 1.

### Preparation of single-cell suspensions

The recently harvested lung specimens were preserved in the sCelLiveTM Tissue Preservation Solution (Singleron Biotechnologies, Nanjing, China) within 30 min of the surgical procedure and promptly transferred to the Singleron laboratory on ice. The specimens were disintegrated into individual cell suspensions using a Singleron PythoNTM Automated Tissue Dissociator (Singleron Biotechnologies) with sCelLiveTM Tissue Dissociation Mix (Singleron Biotechnologies), following the predetermined technique for lung tissues. After trypan blue (Bio-Rad, CA, USA) staining, the samples were evaluated for cell viability using a microscope.

### Single-cell RNA sequencing

Single-cell suspensions with a vitality of above 80% were produced and placed onto microfluidic devices at a concentration of 1 × 10^5^ cells/mL. The scRNA-seq libraries were generated using the GEXSCOPE® Single-Cell RNA Library Kit (Singleron Biotechnologies) following the directions provided by the manufacturer. The libraries of each individual were diluted to a concentration of 4 ng/µL and then combined for sequencing. The Novaseq 6000 (Illumina, San Diego, CA, USA) platform was employed to sequence the pools and generate paired-end reads of 150 bp.

### Quality control, dimension reduction, and clustering

The Scanpy v1.8.1 library was used in Python 3.7 for performing quality checking, dimension reduction, and clustering.The expression matrix of each sample dataset underwent filtering using the following exclusion criteria: (1) cells exhibiting a gene count lower than 200 or falling within the top 2% of gene count; (2) cells displaying a unique molecular identifier (UMI) count within the top 2%; (3) cells manifesting a mitochondrial content below 20%; and 4) Genes expressed in less than five cells. Following the application of filters, typically, 75,613 cells were preserved for downstream analysis, exhibiting an average of 1642 genes and 5266 UMIs per cell. The raw count matrix underwent normalization by dividing the total count per cell and then underwent a logarithmic transformation to produce a normalized data matrix. By setting “flavor = seurat,” we selected the top 2000 variable genes. The scaled variable gene matrix was analyzed using principle component analysis, and clustering and dimension reduction were performed using the top 20 principal components. The Louvain method was used to partition cells into 26 distinct clusters with a resolution value of 1.2. The technique of Uniform Manifold Approximation and Projection (UMAP) was employed to visually represent the clusters of cells^61^.

### Identification of differentially expressed genes (DEGs)

We employed the scanpy.tl.rank_genes_groups function, using the Wilcoxon rank-sum test with default settings, to detect the DEGs. DEGs were identified based on two criteria: genes exhibiting an average log fold change value over 0.25 and genes that were expressed in over 10% of the cells in either comparison group. The Benjamini–Hochberg correction method was used to compute the adjusted p-value. DEGs with an adjusted p-value of p < 0.05 were deemed statistically significant.

### Cell-type recognition using cell-ID

Cell-ID is a method that uses multivariate to identify gene signatures for each unique cell and then classifies cells utilizing hypergeometric tests (HGT)^62^. The normalized gene expression matrix underwent dimension reduction via multiple correspondence analysis. Subsequently, the same low-dimensional space was projected onto both cells and genes. Subsequently, the genes were prioritized, and the predominant gene sets of each cell were determined. Gene sets were subjected to HGT utilizing a brain reference obtained from the SynEcoSys database, which included all the prominent genes from every cell type. The cell type identification was established by selecting the cell type with the lowest HGT p-value. For cluster annotation, we computed the occurrence rate of each cell type inside each cluster and then selected the cell type with the greatest rate as the cluster's identification.

### Functional enrichment analysis

To find the possible roles of DEGs, we conducted Gene Ontology and Kyoto Encyclopedia of Genes and Genomes studies. These analyses were carried out using the "clusterProfiler" R package version 3.16.1^63^. Pathways with p_adj below 0.05 were deemed substantially enriched. The gene set enrichment analysis (GSEA) was conducted employing clusterProfiler (v4.0.0) to identify the gene sets that exhibited substantial enrichment in each individual cell cluster.

### UCell gene set scoring

The process of scoring gene sets was executed utilizing the R package UCell v. 1.1.0^64^. The UCell scores were determined using the Mann–Whitney U test, which included rating the query genes based on their individual cells’ expression levels.

### Cell–cell communication analysis using CellPhoneDB

The interaction between fibroblasts and epithelial cells (ECs) was forecasted based on established ligand-receptor pairings using CellPhoneDB (v2.1.0)^65^. Using 1000 as the permutation number, the null distribution of the average expression of ligand-receptor pairings in randomly assigned cell identities was calculated. The threshold for ligand or receptor expression was established by employing the average logarithmic gene expression distribution for all genes in each cell type. Significance was attributed to interaction pairs that had a p-value below 0.05 and an average log expression greater than 0.1. These significant pairs are graphically represented using the heatmap_plot and dot_plot functions in CellPhoneDB.

### Pseudotime trajectory analysis using Monocle2

The cell differentiation trajectory of monocyte subtypes was reconstructed using Monocle2 v. 2.10.0^66^. The trajectory was constructed by selecting the top 2000 genes with high variability using Seurat (v3.1.2) FindVariableFeatures function and then performing dimension reduction using DDRTree. The plot_cell_trajectory function was employed to illustrate the trajectory.

### Transcription factor regulatory network analysis

A transcription factor (TF) network was constructed using pySCENIC (v0.11.0)^67^ with the scRNA expression matrix and transcription factors in AnimalTFDB^68^. The GRNBoost2 algorithm was used to forecast a regulatory network by analyzing the coexpression patterns of regulators and targets. Subsequently, CisTarget was used to exclude indirect targets and identify transcription factor-binding motifs. Later on, the AUCell was used to measure the level of activity of each neuron in every cell. TFs unique to each cluster were identified based on their regulon specificity scores, and their activity was shown employing heatmaps.

### Hematoxylin–eosin (HE) and Masson staining

HE and Masson staining were separately performed using the pre-prepared paraffin sections (4 μm). HE staining involved dewaxing, rehydration, hematoxylin staining, acid ethanol treatment, distilled water treatment, eosin staining, ethanol gradient dehydration, xylene penetration, neutral gum mounting and observed under a microscope. Masson staining kit (Jiancheng, Nanjing, China) was applied for Masson staining after deparaffinization and rehydration. Fibrosis degrees were observed under a microscope, and images were collected.

### Immunofluorescence staining

Immunofluorescence staining was performed as previously described^69^. After sections were dried, dewaxed, hydrated and underwent high-pressure antigen repairing, the lung tissue sections were incubated at 4 °C overnight with the following primary antibodies: anti-KRT5 (1:100, ab52635, Abcam, MA, USA), anti-VCAN (1: 100, MA5-27638, TermoFisher, MA, USA), anti-COL1A1 (1: 20,000, ab138492, Abcam, MA, USA), and anti-POSTN (1: 2000, ab14041, Abcam, MA, USA). Secondary antibody incubation was performed for 1 h. DAPI dye (AR1176, BOSTER, China) was used to counterstain the nucleus. Confocal images were taken using Leica SP8X Confocal Microscope and the images were processed using ImageJ.

### Statistical analysis

The UCell scores for the scRNA-seq data were compared between the two sets of cells deploying unpaired two-tailed Wilcoxon rank-sum tests. The experimental data were presented as mean ± standard deviation (mean ± SD), and t-test was used for statistical analysis of two group using SPSS software (IBM SPSS 24.0, SPSS Inc). Statistical significance was set at P < 0.05 (*P < 0.05, **P < 0.01, and ***P < 0.001).

### Ethics approval and consent to participate

The research was carried out in compliance with the Declaration of Helsinki and received approval from the Ethics Committee of the Second Affiliated Hospital of Hainan Medical University (No. LW2023162).All participants engaged in the research provided informed consent.

### Supplementary Information

Supplementary Information.

## Data Availability

The single-cell RNA-seq data that provide evidence for the conclusions of this work may be obtained from the corresponding author upon a reasonable request.
