# Supplementary material for: Single-cell RNA sequencing reveals special basal cells and fibroblasts in idiopathic pulmonary fibrosis
Source: Sci Rep. 2024 Jul 9;14:15778. doi: 10.1038/s41598-024-66947-5 (PMC11233624; doi:10.1038/s41598-024-66947-5)
Supplement: Supplementary file 1 — Supplementary Information. [file 41598_2024_66947_MOESM1_ESM.pdf]

Single-cell RNA Sequencing Reveals Special Basal Cells and Fibroblasts in Idiopathic Pulmonary Fibrosis

Chengji Jin[1,†], *Yahong Chen*[1,†], *Yujie Wang*[1], *Jia Li*[4], *Jin Liang*[5], *Shaomao Zheng*[1], *Lipeng Zhang*[4], *Qiaoyu Li*[4], *Yongchao Wang*[6], *Fayu Ling*[7], *Yongjie Li*[7], *Yu Zheng*[4], *Qiuli Nie*[4], *Qiong Feng*[1], *Jing Wang* [1,2,]* & *Huiling Yang*[3,]*

[1]Department of Respiratory Medicine, The Second Affiliated Hospital, Hainan Medical University, Haikou 570100, China
[2]NHC Key Laboratory of Tropical Disease Control, Hainan Medical University, Haikou 571199, China
[3]School of Pharmacy, Guangdong Medical University, Dongguan, 523808, China
[4]The Second Affiliated Clinical College, Hainan Medical University, Haikou 570100, China
[5]Department of Rheumatology and Immunology, The Second Affiliated Hospital, Hainan Medical University, Haikou 570100, China
[6]Singleron Biotechnologies, Yaogu Avenue 11, Nanjing 211800, China
[7]Department of Thoracic Surgery, The Second Affiliated Hospital, Hainan Medical University, Haikou 570100, China

* Correspondence: tlfwj@163.com; 112699355@qq.com
† These authors contributed equally to this work.

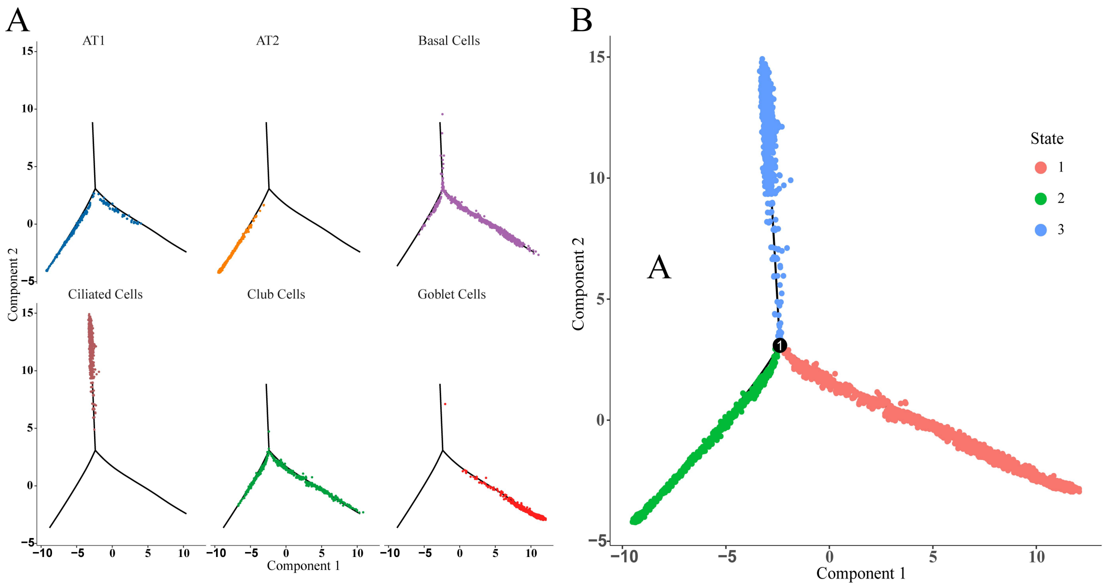

Figure S1. (A) The distribution plot of each individual cell type in trajectory analysis. (B) Trajectory analysis by state.

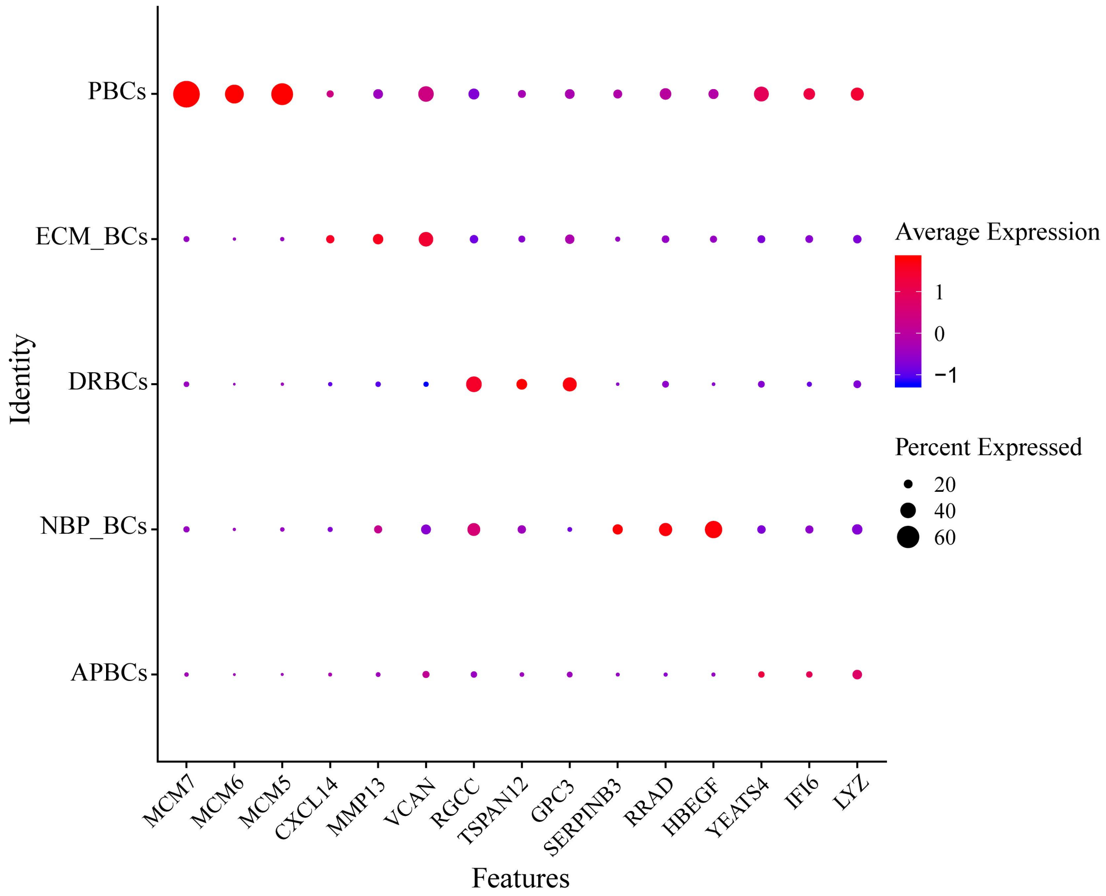

Figure S2. Bubble diagram showing the marker genes in each cell subtypes from basal cells.The bubble size represents the percentage of cells expressing each gene, while the bubble color represents the level of expression.

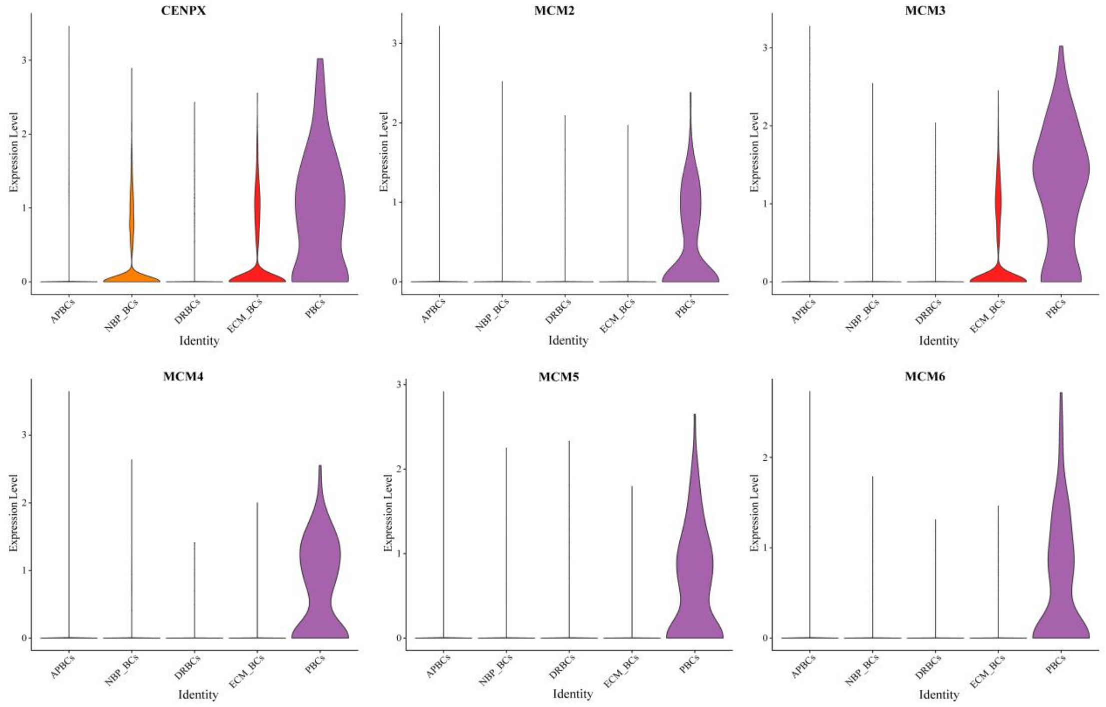

Figure S3. Violin plots present MCM2-MCM7 and CENPX genes differentially expressed.

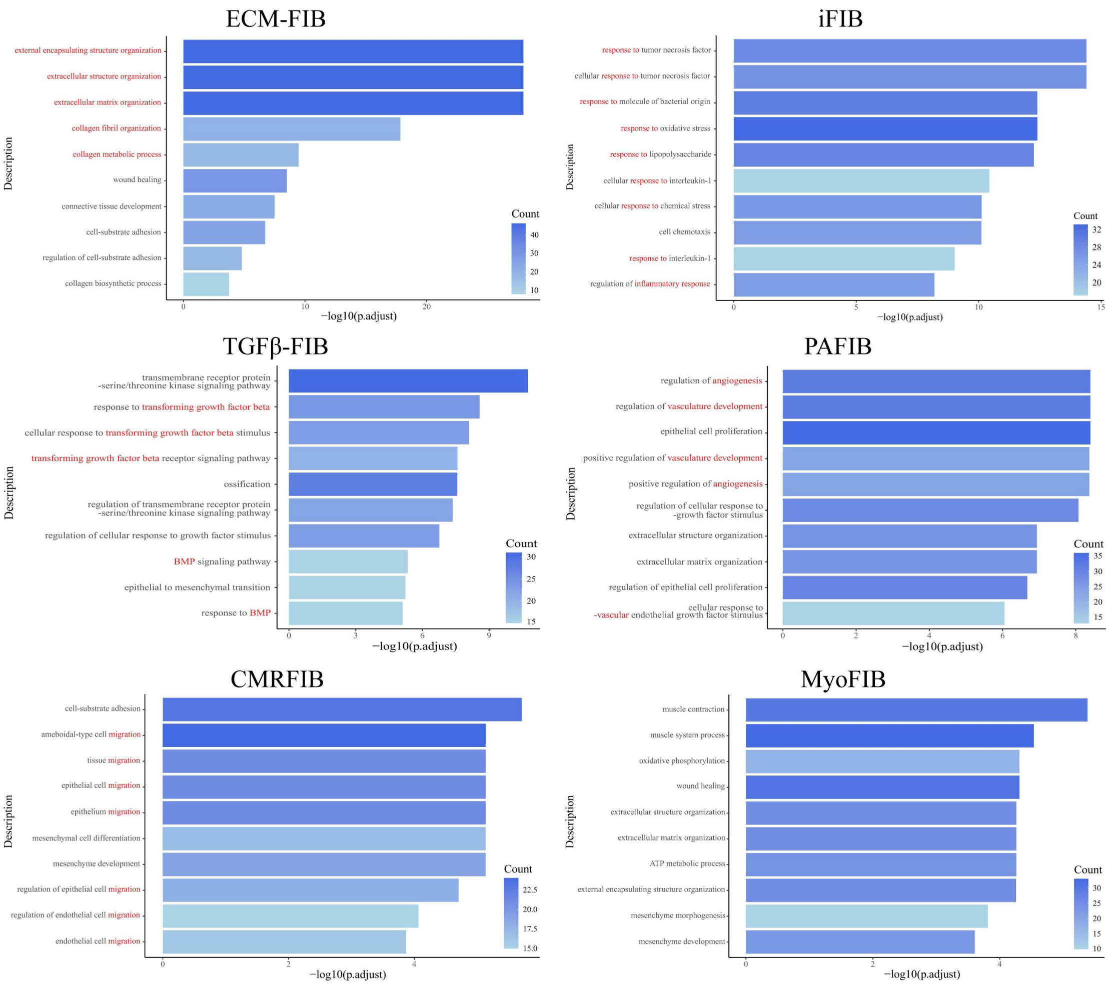

Figure S4. Result of Gene Ontology (GO) Biological Process (BP) enrichment analysis.

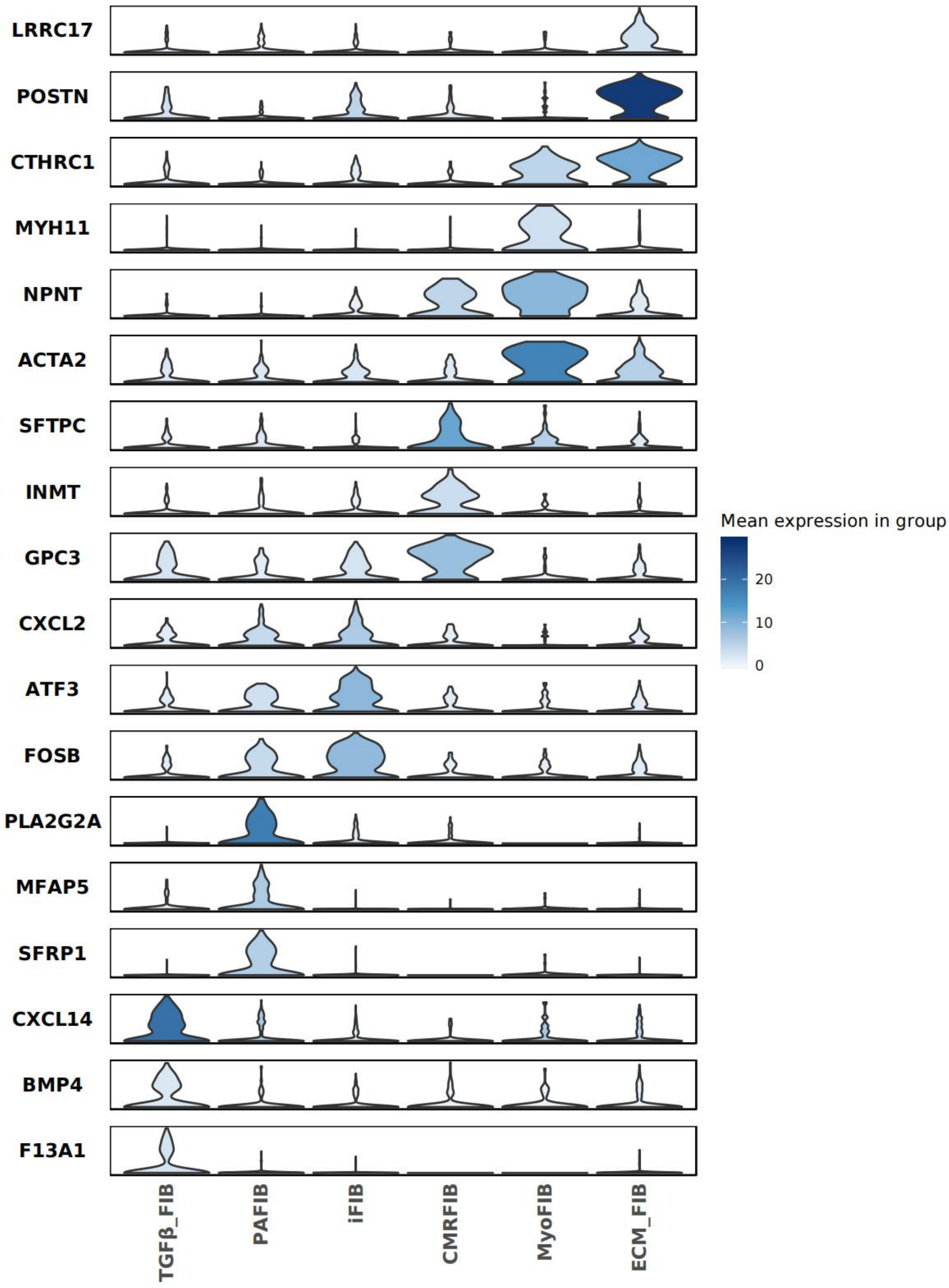

Figure S5. Stacked violin plots displaying markers genes across six subtypes of fibroblasts.

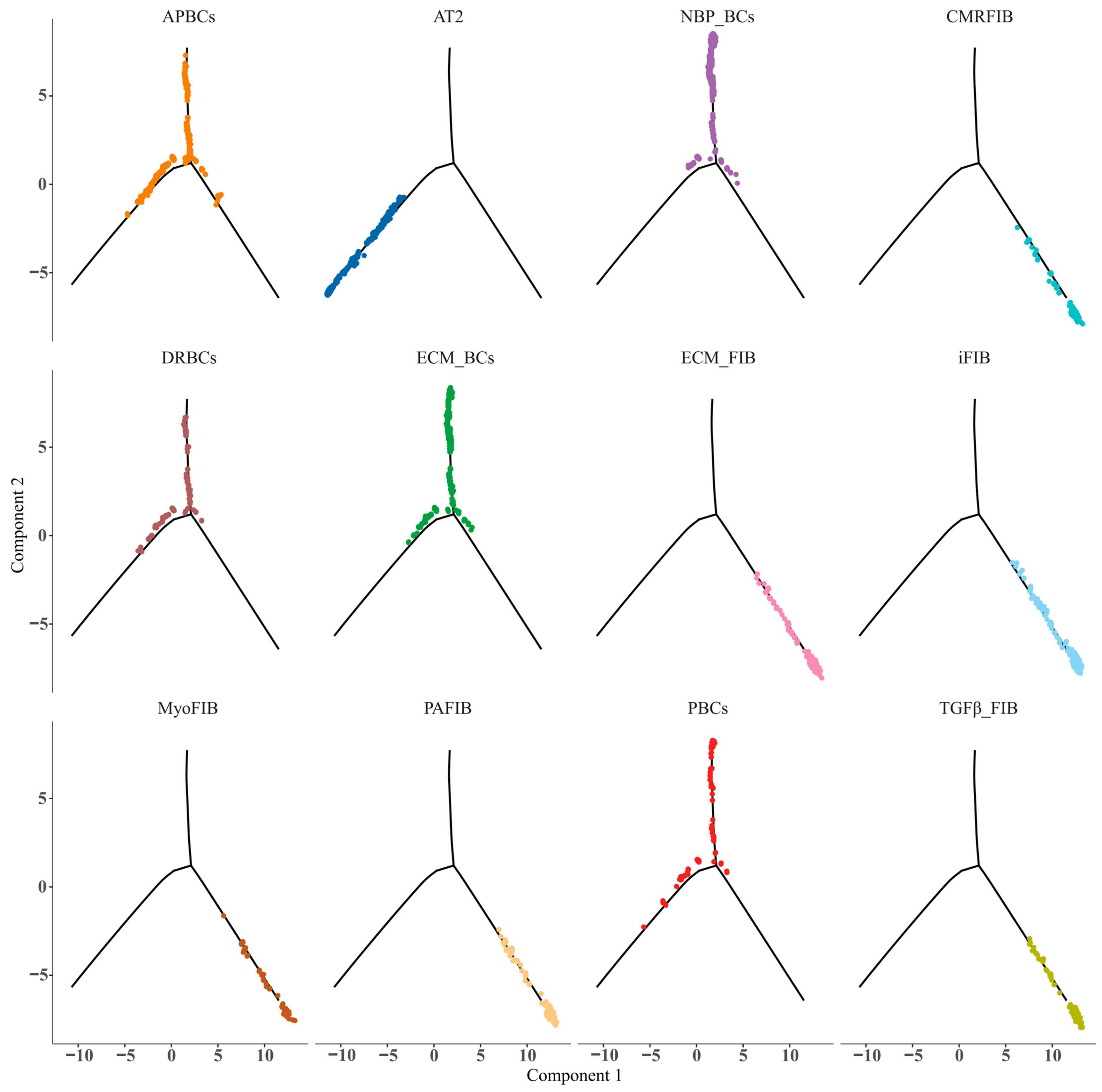

Figure S6. Monocle projection of individual clusters.

Table S1: Patient information for IPF and control samples (n = 16)

Table S1.The basic informations of all patients and controls included in this study.								
Subject ID	Disease Group	Sex	Age	Race	Ever Smoker	sc-RNA-seq	HE Masson	IF
P003	IPF-1	Female	66	Asian	No	Yes	Yes	Yes
P006	IPF-2	Male	66	Asian	Yes	Yes	Yes	Yes
P010	IPF-3	Male	59	Asian	No	Yes	Yes	NO
P013	IPF-4	Male	68	Asian	No	Yes	Yes	Yes
P015	IPF-5	Male	68	Asian	Yes	Yes	Yes	NO
P018	IPF-6	Male	68	Asian	Yes	NO	Yes	NO
P020	IPF-7	Male	68	Asian	Yes	NO	Yes	Yes
P022	IPF-8	Male	59	Asian	Yes	NO	Yes	Yes
C003	Control-1	Male	53	Asian	Yes	Yes	Yes	NO
C004	Control-2	Female	60	Asian	No	Yes	Yes	Yes
C005	Control-4	Female	58	Asian	No	NO	Yes	NO
C006	Control-5	Male	61	Asian	Yes	NO	Yes	NO
C007	Control-6	Female	63	Asian	No	NO	Yes	Yes
C008	Control-7	Male	54	Asian	No	NO	Yes	Yes
C009	Control-8	Male	57	Asian	Yes	NO	Yes	Yes
C010	Control-3	Male	55	Asian	Yes	Yes	Yes	Yes

Notes:

IF: Immunofluorescence

Table S2: Number and proportion of 11 cell types in IPF and control groups.

Table S2.Number and proportion of different cell types				
	IPF		Control	
Celltype	Numbers	Percentage	Numbers	Percentage
Epithelial cells	10321	23.53%	3471	10.93%
Endothelial cells	6902	15.74%	2552	8.04%
Fibroblasts	2015	4.59%	131	0.41%
Mural cells	1608	3.67%	477	1.50%
Proliferating cells	1047	2.39%	326	1.03%
B cells	969	2.21%	4348	13.69%
Plasma cells	185	0.42%	294	0.93%
T and NK cells	10506	23.96%	6856	21.59%
Neutrophils	1086	2.48%	1292	4.07%
Mast cells	348	0.79%	555	1.75%
Mononuclear phagocytes	8870	20.22%	11454	36.07%
Sum	43857	100%	31756	100%

Table S3: Lists of specific markers of the subpopulations in basal cells.

Table S3.Specific markers of the subclusters of basal cells			
Subpopulations	Marker of basal cells	Marker of subtypes	Specific marker
PBCs	KRT5	MCM7	$KRT5^{+}$/$MCM7^{+}$
ECM_BCs	KRT5	VCAN	$KRT5^{+}$/$VCAN^{+}$
DRBCs	KRT5	GPC3	$KRT5^{+}$/$GPC3^{+}$
NBP_BCs	KRT5	HBEGF	$KRT5^{+}$/$HBEGF^{+}$
APBCs	KRT5	LYZ	$KRT5^{+}$/LYZ^{+}

Table S4: Number and proportion of basal cells subclusters in IPF and control groups.

Table S4. Number and proportion of subtype of basal cells				
	IPF		Control	
Celltype	Numbers	Percentage	Numbers	Percentage
APBCs	1571	30.47%	19	51.35%
ECM_BCs	1412	27.39%	4	10.81%
NBP_BCs	1874	36.35%	4	10.81%
DRBCs	187	3.63%	9	24.32%
PBCs	112	2.17%	1	2.70%
Sum	5156	100%	37	100%

Table S5: Lists of specific markers of the subsets in fibroblasts.

Table S5. Specific markers of the subclusters of fibroblasts			
Subpopulations	Marker of basal cells	Marker of subtypes	Specific marker
TGFβ_FIB	COL1A1	CXCL14	$COL1A1^+$/$CXCL14^+$
PAFIB	COL1A1	PLA2G2A	$COL1A1^+$/$PLA2G2A^+$
iFIB	COL1A1	FOSB	$COL1A1^+$/$FOSB^+$
CMRFIB	COL1A1	SFTPC	$COL1A1^+$/$SFTPC^+$
MyoFIB	COL1A1	ACTA2	$COL1A1^+$/$ACTA2^+$
ECM_FIB	COL1A1	POSTN	$COL1A1^+$/$POSTN^+$
